# Role and Importance of Functional Food Packaging in Specialized Products for Vulnerable Populations: Implications for Innovation and Policy Development for Sustainability

**DOI:** 10.3390/foods11193043

**Published:** 2022-09-30

**Authors:** Melvin A. Pascall, Kris DeAngelo, Julie Richards, Mary Beth Arensberg

**Affiliations:** 1 Department of Food Science and Technology, The Ohio State University, Columbus, OH 43210, USA; 2 Institute for Food Laws and Regulations, Department of Food Science and Human Nutrition, Michigan State University, East Lansing, MI 48824, USA; 3 College of Law, Michigan State University, East Lansing, MI 48824, USA; 4 Abbott Nutrition Division of Abbott, Columbus, OH 43219, USA

**Keywords:** functional food packaging, sustainability, medical foods, foods for special dietary use, infant formulas, natural health products, policy development

## Abstract

Specialized products can be needed to help meet the nutrition requirements of vulnerable populations, including infants and young children, those who are ill, and older adults. Laws and regulations delineate distinct categories for such products including medical foods or formulated liquid diets, foods for special dietary use (FSDUs), infant formulas, and natural health products (NHPs). Yet, the literature is limited regarding the role and importance of functional and sustainable packaging for specialized products. This perspective review describes these unique product categories and the role of packaging as well as regulatory considerations. Furthermore, reviewed are how waste reduction strategies and emerging legislative/regulatory policies in the United States and Canada may not adequately address the functional packaging requirements for specialized products. The paper concludes by offering perspectives for emerging innovations and policy development for sustainability.

## 1. Introduction

For products sold direct to consumers, packaging is important for multiple reasons, from helping maintain product integrity to providing information about product use. As environmental concerns continue to drive global actions toward reducing and recycling product packaging—particularly plastic packaging—there is movement to a circular economy where packaging materials are continuously recycled and reused [1]. Yet, in considering how to make packaging more functional and sustainable, food products frequently face greater challenges compared to other types of consumer goods such as household cleaning products. A primary reason is that food packaging must meet stringent requirements to ensure food safety, including limiting the potential for contaminants migrating into foods, regardless of whether the packaging is made with new or recycled materials, and this can be particularly challenging for plastics [2].

Further, maintaining food safety as well as nutrient integrity is especially critical in foods used by vulnerable populations [3], including infants and young children [4], those who are ill [5], and older adults [6,7]. Specialized products can be necessary to help meet the nutrition requirements of these vulnerable populations. The laws and regulations delineate distinct categories for such products including medical foods or formulated liquid diets, foods for special dietary use (FSDUs), infant formulas, and natural health products (NHPs), the category names of which vary based on the country. However, the literature about the role and importance of functional and sustainable packaging for specialized products appears to be limited. In addition, policymakers are failing to consider the potential consequences of putting in place packaging requirements before there are sufficient food safety considerations within the recycling system that are necessary to protect the most vulnerable individuals.

This perspective review describes these unique product categories and summarizes the role of packaging and manufacturing process/regulatory considerations for the packaging of specialized products. The paper also reviews how waste reduction strategies and emerging legislative/regulatory policies in the United States (US) and Canada may not adequately address the functional packaging requirements of these distinctive products. Finally, the paper offers perspectives for emerging innovations and policy development for sustainability, recognizing the challenges faced by specialized products supporting vulnerable populations.

## 2. Specialized Product Use and Regulatory Categorization

To understand packaging’s role and importance in specialized products, it is beneficial to first consider how the products are used and categorized. All humans generally require the same essential nutrients to sustain life; however, individual differences in digestion/absorption, growth/development, clinical condition/disease, and various other factors can impact personal nutrient needs and dietary requirements [8]. Individuals who are part of vulnerable populations, including infants, young children, those with acute and/or chronic conditions/disease, and aging populations may require specialized nutrition products to thrive [9,10]. These products may be the sole source of nutrition or have a targeted role in maintaining nutrition status, underscoring the necessity for the robust regulatory frameworks in place today to help ensure nutrition integrity, quality, and safety. The regulatory frameworks for these specialized products differ somewhat between the US and Canada, though many parallels can be drawn. The following section provides background on the regulatory categorization of products in the US and Canada that support vulnerable populations.

### 2.1. United States

**Figure 1:**
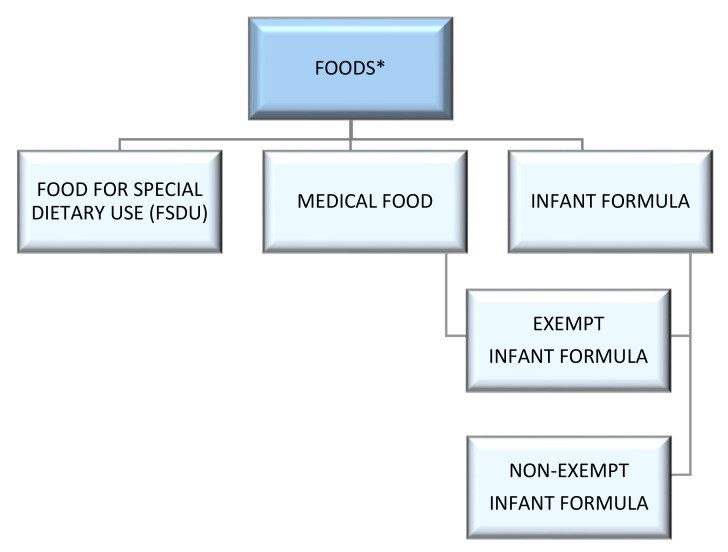
Specialized product categories in the United States. * Foods are regulated by the Food and Drug Administration (FDA) Code of Federal Regulations (CFR).

The US Food and Drug Administration (FDA) is responsible for establishing standards for the safety and quality of foods sold in the US, with the exception of meat, poultry, catfish, and egg products, as those are regulated by the United States Department of Agriculture (USDA). The FDA has established governing regulations for several different specialized food categories within its jurisdiction (Figure 1). These regulations dictate requirements for nutrition, labeling and claims, and standards for food safety and quality, including good manufacturing practices (GMPs). While all foods must meet the same basic standards, several categories have additional regulations or exemptions due to their nature and use. These include medical foods and infant formulas.

The FDA views medical foods, FSDUs, and infant formulas as separate and distinct categories (Figure 1) in part based on the legislative origin for the categories. Medical foods are a subset of foods and are defined in the Orphan Drug Act, 21 USC 360ee(b)(3), as “a food which is formulated to be consumed or administered enterally under the supervision of a physician and which is intended for the specific dietary management of a disease or condition for which distinctive nutritional requirements, based on recognized scientific principles, are established by medical evaluation” (Table 1) [11]. Medical foods are distinguished from the broader category of FDSUs based on “the requirement that medical foods be intended to meet distinctive nutritional requirements of a disease or condition, used under medical supervision, and intended for the specific dietary management of a disease or condition” [12]. Thus, it is important to note that not all foods fed to patients with a disease are medical foods. Instead, medical foods are specially formulated and processed for a patient who requires use of the product as a major component of a disease or condition’s specific dietary management [12]. In FDA’s “Frequently Asked Questions About Medical Foods; Second Edition Guidance for Industry,” FDA notes that medical foods designed to meet the distinctive nutritional requirements of individuals with inborn errors of metabolism (IEMs) are an example of medical foods required in the specific dietary modification of these patients [12]. Further, in FDA’s Compliance Program Manual for Medical Foods, FDA articulates that medical foods can be classified into the following categories: nutritionally complete formulas, nutritionally incomplete formulas (including individual modular type products that may be mixed with other products before use), formulas for metabolic (genetic) disorders in patients over 12 months of age, or oral rehydration products [13].

In contrast, FSDUs make up a larger category of foods. The FDA defines the term “special dietary uses” in 21 CFR 105.3, as “particular (as distinguished from general) uses of food, as follows: (i) Uses for supplying particular dietary needs which exist by reason of a physical, physiological, pathological or other condition, including but not limited to the conditions of diseases, convalescence, pregnancy, lactation, allergic hypersensitivity to food, underweight, and overweight; (ii) Uses for supplying particular dietary needs which exist by reason of age, including but not limited to the ages of infancy and childhood; (iii) Uses for supplementing or fortifying the ordinary or usual diet with any vitamin, mineral, or other dietary property. Any such particular use of food is a special dietary use, regardless of whether such food also purports to be or is represented for general use” (Table 1) [14]. FSDUs are not specifically formulated to be administered under the supervision of a physician, though a physician may recommend an FSDU product. An example of an FSDU is an oral nutrition supplement (ONS), which is a manufactured liquid, reconstitutable powder, or solid product that contains a combination of carbohydrates, proteins, fats, fiber, vitamins and/or minerals intended to supplement a portion of an individual’s nutrition intake [17].

Infant formula is another distinct category of food. Infants are defined as those under 12 months of age (21 CFR 105.3(e)) [14]. Congress enacted special provisions in the *Infant Formula Act of 1980* that define infant formulas as “a food which purports to be or is represented for special dietary use solely as a food for infants by reason of its simulation of human milk or its suitability as a complete or partial substitute for human milk” (21 USC 321(z)) [15].

Infant formula is categorized as either non-exempt or exempt depending on the intended infant’s nutritional needs (Table 1). A non-exempt infant formula is one meant for healthy, term infants and represents the majority of infant formula products fed. Exempt infant formula is defined in 21 CFR 107.3 as “an infant formula intended for commercial or charitable distribution that is represented and labeled for use by infants who have inborn errors of metabolism or low birth weight, or who otherwise have unusual medical or dietary problems” [14], Human milk fortifiers follow exempt infant formula regulations (21 CFR 107.5) [18] because they are intended for premature or low birth weight infants.

Because medical foods, FSDUs, and infant formulas remain classified as foods, the laws and regulations governing foods apply, unless otherwise stated in laws/regulations. It is imperative that manufacturers register food facilities with the FDA, follow current GMPs, and establish Hazard Analysis and Critical Control Practices (HACCP), and Hazard Analysis and Risk Preventive Control (HARPC) procedures among other standard food provisions under the Food Drug and Cosmetic Act (FD&C Act) and Food Safety Modernization Act (FSMA) to help ensure food safety. Moreover, allergen labeling rules must be followed under the Food Allergen Labeling and Consumer Protection Act of 2004 (FALCPA). Some manufacturers also follow voluntary standards such as Safe Quality Food (SQF), and those issued by National Standard Foundation (NSF), International Organization for Standards (ISO), and ASTM (formally known as American Society for Testing Materials). The requirements demanded by these many standards are designed to ensure that no adulterated or misbranded food products are manufactured, stored, transported, sold, or received into commerce within the US and Canada.

There are additional statutory and regulatory requirements for specialty foods beyond those that apply to conventional foods. For example, for infant formula, FDA has established compositional and labeling requirements; infant formula GMPs, which include testing requirements prior to release into distribution; requirements for manufacturers to demonstrate specific quality factors for an infant formula, including demonstration of normal physical growth and sufficient biological quality of protein; and requirements for pre-market notification of any new or changed infant formula, which includes a review of the infant formula packaging materials. FDA has not established specific labeling criteria for medical foods, to date. However, medical foods are targeted to certain disease states/conditions, and as such, they are exempt from the conventional food labeling requirements in 21 CFR 101.9 and other provisions that regulate the broader category of foods. FDA has established specific definitional criteria for medical foods, among which is the requirement that they be used under active and ongoing medical supervision. Therefore, medical foods are typically labeled with “use under medical supervision”.

### 2.2. Canada

**Figure 2:**
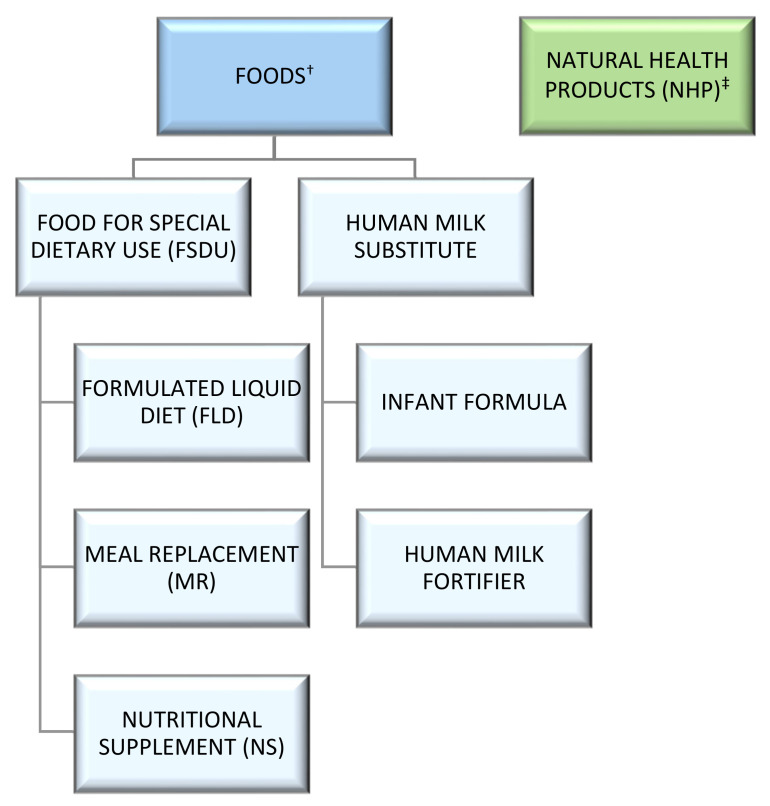
Select * specialized product categories in Canada. * As discussed in this paper. ^†^ Foods are regulated by Health Canada’s Food and Drug Regulations (FDR). ^‡^ Natural Health Products are regulated by Health Canada’s Natural Health Product Regulations (NHPR); NHP categorization depends on characterization, formulation, history, and public perception.

Health Canada is the government department responsible for establishing standards for the safety and quality of foods and NHPs sold in Canada. Food manufacturers are required to follow the regulations described in the Food and Drug Regulations part B [19], and NHP manufacturers are required to follow the regulations described in the Natural Health Product Regulations [20]. Figure 2 outlines Canada’s regulatory categories discussed in this paper.

Under the Food and Drug Act of 1985, food is defined as “any article manufactured, sold or represented for use as food or drink for human beings, chewing gum, and any ingredient that may be mixed with food for any purpose whatever” [21]. There are specialty categories that are afforded additional regulations, including food for special dietary use (FSDU) which is defined as a “food that has been specially processed or formulated to meet the particular requirements of a person (a) in whom a physical or physiological condition exists as a result of a disease, disorder or injury, or (b) for whom a particular effect, including but not limited to weight loss, is to be obtained by a controlled intake of foods” (FDR B.24.001) [22].

As prescribed in the regulations, FSDUs in Canada include namely formulated liquid diets (FLD), meal replacements (MR), and nutritional supplements (NS) (Figure 2). The FDR defines the FLD category as “a food that (a) is sold for consumption in liquid form, and (b) is sold or represented as a nutritionally complete diet for oral or tube feeding of a person described in paragraph (a) of the definition ‘food for special dietary use’” (FDR B.24.001) [22]. Thus, FLDs are often the sole source nutrition product for a person with a physical or physiological condition, and FLDs can be administered orally or via tube feeding. The MR category is defined as “a formulated food that, by itself, can replace one or more daily meals” (FDR B.24.001) [23]. The NS category contains “a food sold or represented as a supplement to a diet that may be inadequate in energy and essential nutrients” (FDR B.24.201) [23]. Each of the FSDU categories have specific compositional requirements to help meet the specific nutritional needs of their respective intended users (Table 2).

The Canada Food and Drug Regulations also include definitions for other categories, similar to regulations in the US. The human milk substitute category includes both infant formulas and human milk fortifiers (Table 2). This category consists of infant food, which is “a food that is labelled or advertised for consumption by infants” [24]. Similar to the US, in Canada an infant is defined as “an individual who is under the age of one year” [24].

Much like infant formula in the US, infant formula in Canada must meet specific compositional requirements as defined by Health Canada in Part B Division 25 of the FDR [24]. There are human milk substitute labeling requirements, specific GMPs, including testing criteria, and requirements to submit a pre-market notification to Health Canada prior to introduction to the market of a new human milk substitute or one that has undergone a major change. Pre-market notification includes evidence of acceptable growth and development, as well as a review of the packaging.

Natural Health Products (NHPs) are regulated by Health Canada’s Natural Health Product Regulations and are defined as “a substance … that is manufactured, sold or represented for use in (a) the diagnosis, treatment, mitigation or prevention of a disease, disorder or abnormal physical state or its symptoms in humans; (b) restoring or correcting organic functions in humans; or (c) modifying organic functions in humans, such as modifying those functions in a manner that maintains or promotes health” [20]. These products include but are not limited to vitamins and minerals, amino acids, essential fatty acids, probiotics, herbal remedies, and oral rehydration solutions. Unlike foods, NHPs are not intended to be used as a primary source of nourishment [25]. However, the line between an NHP and food often blurs because many components which are traditional ingredients in food (including vitamins, minerals, probiotics, herbs, amino acids, and fatty acids) when sold individually or in combination may be labeled NHPs depending on the product composition, representation, history of use, and public perception.

## 3. Role of Functional Packaging for Foods and Specialized Products

Food packaging has emerged as both a functional and essential tool for the preservation, containment, transportation, storage, and marketing of food, plus it can provide information about the product it contains. Functional packaging provides value-added benefits such as greater convenience and ease of use, while essential packaging elements include those critical for food quality and safety. The FDA and USDA regulate food packaging in the US. In Canada, the responsible regulatory agency for food packaging is Health Canada [26]. Further details on the role of food packaging, particularly the implications for specialized products, are described below.

### 3.1. Preservation and Protection

Packaging is critical for preserving the safety, quality, and nutrient integrity of food or specialized product throughout the life of the product. Protection is also paramount, as packaging provides a barrier to biological, chemical, and physical hazards. It is estimated that 1 in 6 Americans [27] and 1 in 8 Canadians [28] are affected by a foodborne illness annually. Hermetically sealed food packaging provides a barrier against microorganisms, including pathogens and spoiling agents that could lead to foodborne illness, and against insects and animals. Preservation and protection are imperative for the foods and specialized products discussed in this paper, since these compositionally complex, shelf-stable products often have an extended shelf-life of greater than 12 months prior to opening.

In addition to protecting products from outside contaminants, packaging acts as a barrier to reduce compositional changes that can result from environmental influences, such as exposure to oxygen, moisture, or light. Packaging also protects the product from damage that could occur during distribution. Physical protection can help maintain the quality of the product for further use, such as a resealable lid/cap. Thus, food packaging helps to limit product degradation, maintain nutrient stability, and preserve overall quality, which are all critical for products that provide essential nutrition to vulnerable populations.

### 3.2. Containment and Food Waste Reduction

In today’s intricate supply chains, containment and waste reduction are important roles of packaging. It is estimated that about 17% of global food production is wasted, with 11% of that number coming from household waste [29]. Packaging can help reduce food waste by extending the shelf life and prolonging the product’s usability [30]. Resealable features on packaged food products can help extend usability, as these features can allow for multiple servings from the same container and may help prevent the unused product from being thrown away immediately. Packaging optimization strategies can help fight against food waste, yet there is also a need for education on how to better utilize packaged food products to prevent waste [31], which is important for specialized products too. Individuals are frequently unaware of the importance of appropriate packaging to reduce food loss and waste, and they often associate a food product’s sustainability with minimal packaging rather than with packaging that keeps food fresh longer [32].

### 3.3. Transportation and Traceability

Packaging is needed for products to be successfully transported and can be beneficial for traceability too. Traceability is extremely important in maintaining safety and authenticity of the product [33]. In the US, after the passing of the FSMA in 2011 (21 USC 111-353), the FDA can now require manufacturers to have processes in place for full end-to-end traceability and for transmitting required data elements for designated high-risk foods [34]. The FDA’s *Blueprint for the New Era of Smarter Food Safety* [35], released in 2020, outlines the intended action the FDA plans to take regarding tech-enabled traceability to quickly identify the source of an outbreak and to remove affected product from store shelves [36]. Food safety and food traceability requirements for foods marketed and sold in Canada are outlined in the Safe Food for Canadians Act and corresponding regulations, which are based on the international standards established by Codex Alimentarius [37,38]. In both the US and Canada, food manufacturers incorporate unique lot codes on packaging. This coding can be presented in various formats. Coding allows manufacturers to track products through all stages of the supply chain, and it allows individuals to determine if foods purchased are affected by a product recall.

### 3.4. Storage, Convenience, and Integrity

Product users often value the storage, convenience, and integrity afforded by packaging. Ease of opening and resealability are packaging features that may be particularly key for those purchasing specialized products like infant formula and similarly categorized products. These specialized products typically come in two different forms—powdered or liquid (either ready-to-use/feed or concentrated liquid). Powdered and concentrated liquid products require reconstitution with water prior to feeding.

Ready-to-feed infant formula is the most convenient form since it requires no preparation. However, powdered infant formula is the predominant form purchased [39] and typically has a longer shelf-life. Manufacturers usually sell powdered infant formula in plastic tubs or metal cans that are resealable, allowing for multiple servings. Packaging convenience factors like resealability can reduce the amount of packaging and food waste. Additionally, most powdered infant formula products come with a plastic scoop, also a part of the packaging, which makes preparation easier for consumers and can help improve accuracy and consistency of preparation.

Manufacturers may incorporate packaging security features to support product integrity and combat tampering, bioterrorism, or product counterfeiting [33]. Some tamper-evident features include banding, breakaway closures, or special printing on bottle liners that irreversibly change upon opening [30]. A US National Retail Foundation’s 2020 survey on organized retail crime listed infant formula as accounting for 13% of the top stolen items from American stores [40]. Counterfeit infant formulas are also a growing concern. Checking containers for damage prior to use can help protect infants from being fed compromised or counterfeit formula that could cause serious adverse health consequences [41]. Because tamper-evident packaging can require additional materials, this safety feature may exacerbate disposal issues, but the benefits generally outweigh the drawbacks [30]. This risk calculation is especially true when the product is being provided to a vulnerable population.

### 3.5. Marketing and Product Information

Prior to purchase, a product’s packaging may be one of the first ways consumers are exposed to or learn about a product. The package design, functionality, and appeal can all inform consumers as they make decisions about products that best meet their needs. Indeed, when shopping for foods, consumers often base purchase decisions on extrinsic product characteristics and appearance [42]. For vulnerable populations like older adults there is evidence that packaging itself can be a potential barrier to adequate nutrition when it is not “easy to open” [43].

Additionally, the package bears the label, which contains critical information for safe and effective product use, especially important for specialized products for vulnerable populations. In every country, there are local requirements related to the labeling of product identity, nutrition information, ingredients, allergens, net weight, manufacturer, and other information. There is also a growing interest in including package disposal information/instructions, such as recyclability and/or post-consumer recycled content (PCR) on package labels.

Clear, accurate information is particularly important for the specialized products discussed in this paper, as many individuals are purchasing these products to help fulfill a nutrition need that may not be met through a normal diet and traditional food alone. Infant formula has very specific labeling requirements in the US and Canada, many of which are related to nutrition information as outlined in Subpart B of 21 CFR 107 [44] and FDR B.25.057(1) [45]. On infant formula labels, directions for preparation and use are also required to ensure safe and correct feeding practices. Clear, precise instructions on powdered infant formula are critical for increased accuracy in infant formula preparation [46]. Moreover, inaccurate infant formula preparation can lead to negative health outcomes such as altered infant growth or infant adiposity [46]. Package design and size can help ensure that nutrition-related information, directions for safe preparation and use, and all other pertinent information is included on the package in an easy-to-understand way.

## 4. Packaging Considerations for the Manufacturing Process and Applicable Regulations

The main material types used to fabricate food packaging include metal, glass, plastic, paper, and composites made from combinations of more than one material type. Several factors go into the choice of material for a given package. These include but are not limited to the nature of the product, its nutritional content, factors that affect its shelf life, the targeted end user, storage requirements, consumer perceptions, and convenience. Indeed, selection of packaging materials appropriate to a product and its intended use is an integral part of processing operations. Consideration must also be given to how packaging materials meet applicable regulatory requirements.

Many specialized products are nutrient-dense, consumed orally or by enteral feeding tube, and may be available in ready-to-use forms (or concentrated forms requiring minimum preparation) as either liquids or powders. The products are manufactured under GMPs and following sanitation standard operating procedures. All packaging materials must be able to withstand the requisite processing and heat treatment conditions and the packaging must keep the product safe and protect against potential chemical compound migration from any packaging materials in direct contact with the specialized products. These characteristics along with the role of maintaining product nutrient levels to support the nutrition of vulnerable populations place very high requirements and expectations on the packaging for specialized products.

### 4.1. Manufacturing for Specialized Products

Several technologies can be used to process specialized products for vulnerable populations. Since these products tend to have higher nutrient content than processed foods for the general population, the preparation and packaging methods selected must not degrade the concentrations of nutrients. Failure to maintain nutrient concentrations at levels on a product’s label could render the product misbranded under the FD&C Act. In the case of infant formula, the product could be deemed adulterated if it fails to contain mandatory nutrients at the required concentrations [47]. More importantly, product nutrient levels must be maintained to help meet the nutrition needs of the vulnerable groups that depend on these products.

In addition to nutrition considerations, the selected processing method must adequately treat the product to ensure its quality and safety from microbial contamination. Packaging material requirements can vary depending on the type of product and manufacturing process, with processes involving high heat or pressure placing the most strenuous demands on packaging materials (Table 3). For specialized products in liquid form, one of three manufacturing processes are used—retort, hot fill, or aseptic—to produce commercially sterile products that are shelf-stable for an extended period without refrigeration. An example of the definition of commercial sterility is found in FDA regulations specific to aseptic packaging, where the FDA specifies “’commercial sterility’ of equipment and containers used for aseptic processing and packaging of food means the condition achieved by application of heat, chemical sterilant(s), or other appropriate treatment that renders the equipment and containers free of viable microorganisms having public health significance, as well as microorganisms of non-health significance, capable of reproducing in the food under normal nonrefrigerated conditions of storage and distribution” [48].

Retorting is a heat treatment method used to sterilize packaged foods, including low-acid specialized products. In this technique, the product is packaged and sealed within the container before heated under pressure at a given temperature and time regimen that is sufficient to create commercial sterility [52]. Several types of retorting systems are currently in use in the food industry, including still and agitated retorts. Irrespective of the retorting types used to process the food, the intent is to manufacture a product that is commercially sterile within a hermetically sealed package. Retort processing of low-acid foods is rigorously monitored by the FDA and it also requires preapproval and certified operators. Packaging types used for retort-processed products can include metal cans, glass and plastic bottles, multilayer pouches, and multilayer composite drink cartons [53].

Hot fill or pasteurization is a different heat treatment technique that can be used to safely manufacture high-acid liquid foods. In this process, the product is sterilized but packaged in non-sterile containers. This process contrasts with aseptic processing in which both the product and the package must be sterilized. However, the pasteurization process must be sufficient enough that the heat of the product or sanitization of the package kills any pathogens.

Aseptic processing can be used for both low and high-acid foods. Low-acid products are generally aseptically processed using ultra high temperature (UHT) methods since this technique has been shown to create commercial sterility within these products with minimal loss of nutrient and sensory properties [54]. Sterilization methods used in aseptic processing include steam, radiation (gamma, e-beam, ultraviolet light, infrared energies), microwave, ohmic, high pressure, and pulse light technologies. Some of these technologies are in commercial use, while others are under research as new innovative methods. Irrespective of the selected technology, it is essential that an appropriate packaging is chosen and be hermetically sealed after filling. Further, its integrity must be maintained before being opened by a consumer.

During aseptic processing and packaging of low-acid foods, the entire system and procedures must be preapproved by the FDA before being employed to manufacture products. This approval includes the equipment construction and material selection, the food treatment method (such as temperature and time in the case of steam treatment), monitoring devices, certification of the operators, quality control testing, record keeping, audits, packaging material and equipment sterilization, and clean-in-place procedures (FDA, 2014). In an aseptic system, the product is sterilized and then packaged and sealed in sterile containers within a sterile environment. The sterile conditions must be maintained during the entire production run. If sterility is lost at any time, the product must be discarded or reprocessed, and the equipment re-sterilized before the packaged product is allowed into retail marketing [52]. Packaging types used for aseptically treated products can include metal cans, glass and plastic bottles, multilayer composite drink cartons, and multilayer flexible laminations [53].

For both packaged retort- and aseptic-treated products and hot-fill high-acid foods, it is necessary to ensure that the integrity of the container is maintained throughout the manufacturing and distribution process. This integrity is fundamental to product safety because there is no refrigeration, freezing, or chemical treatment for long term storage—product safety is maintained by the package. Thus, the package is the sole barrier against contamination of the product after it is processed and sealed within the container. Containers with defective seals are vulnerable to re-contamination. As a result, it is essential to perform testing procedures (destructive and non-destructive) to ensure that the seals of these packages are robust enough to handle palletization, warehousing, transportation, and other environmental conditions and stresses without compromise. In addition, seal integrity can be determined by simulations or actual road testing [55].

Specialized products are also processed and marketed in powdered forms. Current technology does not exist for production of commercially sterile powdered products. While the water activity of these products is too low to support the growth of microorganisms, the products can still be contaminated with microbial spores. It is thus essential that powdered products are made using ingredients with rigorous quality and safety standards and that GMPs are practiced during all aspects of the production run. Although powdered formulations are not sterile, there is still an expectation from the regulators for ensuring product quality and safety (especially for infant foods) and that manufacturers must take steps to “ensure that nutrient levels required by 21 CFR 107.100 (Infant Formula) are maintained in the formula, and the formula is not contaminated with microorganisms or other contaminants” (21 CFR 106.50) [56]. This expectation requires that “each container of finished product is properly sealed” (21 CFR 106.50) [56]. Similarly, Health Canada has established requirements to ensure hermetically sealed low-acid canned foods have proper seal and package integrity and meet commercial sterility requirements (B.27.002(1) and B.27.003) [57].

### 4.2. Packaging Materials for Specialized Products

Historically, packaging for specialized products was typically composed of metal or glass. Steel was often the metal material of choice since it was tough and resilient to heat and pressure. However, because it was also susceptible to corrosion from moisture, steel cans for specialized products were typically coated with various polymers to maintain product safety and quality. Glass used in food packaging has been called soda-lime because it was made with sand (silicon dioxide), limestone (calcium carbonate), and sodium carbonate as the predominant ingredients. Glass is inert and exhibits little reaction with most foods. Thus, there is little absorption of flavors from foods and there is almost no migration of chemicals from the glass to foods. Glass is also impermeable to gases and vapors, and it provides foods with long shelf life due to its tough and heat resistant nature. The main disadvantage of glass is its susceptibility to breakage. It is also more expensive than other types of packaging materials and heavier in weight. In addition, the process of glass package manufacturing is more energy intensive, resulting in greater release of greenhouse gas emissions compared to other packaging manufacturing.

In more recent decades, packaging for specialized products has shifted to include multilayer composites (drink cartons), multilayer flexible pouches, and semirigid plastic bottles. All these materials must meet the functional packaging roles described earlier in this paper as well as the rigorous manufacturing process requirements of specialized products for vulnerable populations (Table 3). Due to its chemical structure, plastic is the most versatile material used to fabricate packaging [58], including for specialized products. Plastic is a polymer made of long chain carbon to carbon atoms bonded together with different types of functional groups attached to it in head-to-tail arrangements. These functional groups can be substituted by a variety of other organic molecules. Since the properties of the resultant polymer are influenced by the type of functional group attached to the polymeric chain, the types of plastics that can be synthesized are almost limitless [59]. For example, polyethylene (PE) is made from a carbon-to-carbon chain having hydrogens in a head to tail arrangement. If the hydrogen is substituted with methyl groups, polypropylene (PP) is produced with different thermal properties such as a higher melting point, and different mechanical properties such as increased tensile strength. Because PP is able to withstand both high heat and high pressure it is frequently used in retort and hot-fill manufacturing of specialized products. Another type of petroleum-based plastics used in food packaging for specialized products is polyethylene terephthalate (PET), however it is typically used in aseptic manufacturing. Plastic packaging can be made from a single polymeric material (such as PET, PP, high density polyethylene (HDPE), and low density polyethylene (LDPE)) or from multilayers, which are combinations of several types of plastic layers [60].

### 4.3. Packaging and Food Contact Substance Regulations

In addition to the packaging being appropriate for manufacturing requirements, packaging must also be safe—particularly for products used by vulnerable populations. Packaging may inadvertently cause chemical, microbiological, or other contamination. It is therefore critical to understand both the potential for and magnitude of such risks when considering packaging options for use in medical foods or formulated liquid diets, FSDUs, infant formulas, and NHPs. It is important to recognize that food contact substances may potentially negatively affect sensitive individuals at an increased rate (compared to a more general population) due to an individual’s age, size, metabolism, or health conditions.

In the US, a food additive is defined as “any substance the intended use of which results or may reasonably be expected to result, directly or indirectly, in its becoming a component or otherwise affecting the characteristics of any food (including any substance intended for use in producing, manufacturing, packing, processing, preparing, treating, packaging, transporting, or holding food)” (21 USC 321(s)) [61]. FDA considers any material used in the production of food packaging as subject to the definition of food additive only if it may reasonably be expected to become a component of or to affect the characteristics of the food packed in the container (21 CFR 170.3(e)(1)) [62]. However, this is largely not the case for food packaging materials. If there is no migration of a packaging component from the package to the food, FDA does not consider it as becoming a component of the food and thus is not a food additive (21 CFR 170.3)) [62].

The FD&C Act deems packaging materials as food contact substances (FCSs) under the definition of “any substance intended for use as a component of materials used in manufacturing, packing, packaging, transporting, or holding food if such use is not intended to have a technical effect in such food” (21 USC 348(h)(6)) [63]. Regardless of the various provisions that define packaging, the penultimate requirement as dictated by Congress with the Food Additive Amendment of 1958 is that packaging materials for food must be safe.

To determine what is “safe”, FDA looks to a reasonable certainty in the minds of trained scientists that the substance is not harmful under the intended use, including the probable consumption, cumulative effect, and other factors deemed salient by qualified experts (21 CFR 170.3(i)) [62]. This determination is particularly important for vulnerable populations because these individuals may have a limited diet, which in some cases may be entirely composed of a single type of food. Such limitations can occur because of few alternatives, tolerance issues, reluctance to try other types of foods, or other nutrition reasons that led to their use of specialized products.

However, FDA does not test packaging to determine safety, rather this is the responsibility of the manufacturer. There are several methods accepted by the FDA for determining if packaging is safe for food contact use, including packaging that may be used for specialized products. First, a manufacturer may submit a food contact notification (FCN) pursuant to 21 USC 348 (h) [64]. This procedure gives FDA 120 days to review the use of an FCS in a specific food by a manufacturer. If FDA does not object to the safe use of the FCS, the company can legally use the FCS for that food. The FCN includes a comprehensive summary, chemical identity, intended conditions of use, intended technical effect, estimation of dietary intake, toxicity information, and environmental information [65].

An FCN is typically preferred by industry due to the rapid time it allows from design to use. However, there are situations in which a lengthier petition must be submitted under 21 CFR 171.1 to authorize the safe use of an FCS. A food additive petition (FAP) process may be required by FDA: (i) when the use of the food contact material will increase the cumulative estimated daily intakes (CEDI) of the substance from both food and food contact uses to a level equal to or greater than 1 ppm, assuming the substance is not a biocide; (ii) for a substance that is a biocide if the CEDI is increased to a level greater than 200 ppb; or (iii) when existing data for the substance include one or more bioassays that FDA has not reviewed and are not clearly negative for carcinogenicity (21 CFR 170.100(c)) [62].

Another potential method for documenting safety of an FCS is by a previous Generally Recognized as Safe (GRAS) classification. Eligibility for classification as GRAS is “based on only the views of experts qualified by scientific training and experience to evaluate the safety of substances directly or indirectly added to food. The basis of such views may be (1) either scientific procedures or (2) in the case of a substance used in food prior to January 1, 1958, through experience based on common use in food. General recognition of safety requires common knowledge throughout the scientific community knowledgeable about the safety of substances directly or indirectly added to food that there is reasonable certainty that the substance is not harmful under the conditions of its intended use” (21 CFR 170.30(a)) [62]. The general recognition of safety for GRAS substances or substances used in food prior to 1958 requires “the same quantity and quality of scientific evidence as is required to obtain approval of the substance as a food additive” [66].

In Canada, a “package” includes anything in which any food, drug, cosmetic or device is wholly or partly contained, placed, or packed (Food and Drugs Act) [21]. Packages are specifically excluded from the definition of food additives and regulated separately under Division 23 Part B of the Food and Drugs Regulations. Regardless, the Food and Drugs Act and corresponding regulations prohibit the sale of food in packages that may impart any harmful substances that may be injurious to consumers (FDR B.23.001) [67]. To this extent, and unlike the US, there are lists of substances, finished articles, and components, which are permitted to be used in food packaging materials in Canada. In addition, Health Canada maintains lists of equivalent polymer resins, available upon request, which may be used interchangeably [68].

However, for substances that are not already approved, packaging materials may be submitted to the Food Directorate’s Health Products and Food Branch (HPFB) on a voluntary basis (except infant formula and HMF which require a submission) for a premarket assessment and ruling on chemical safety [26]. Manufacturers may submit both finished products and individual components for an assessment of safety. The notification letter must include name, full composition (which includes quantitative listing of all components, with each listed by proper chemical name and/or trade name, grade, and supplier), and intended use. Other information that may be submitted includes material safety data sheets, technical data sheets, product literature, migration data, extraction data, and toxicological data. After full information is obtained, HPFB may issue a Letter of No Objection (LONO) with limitations, if any, or may request further information [69]. LONOs are valid into perpetuity unless the composition or intended use change. Most importantly, because LONOs are not approvals of a particular component per se, Health Canada is permitted to rescind a LONO status if new scientific data becomes available [26].

Finally, like the US, Canada has promulgated special rules related to infant formula packages and FSDU packaging including strict adherence to GMPs, use, sales, labeling, directions, etc., as well as non-binding guidelines for determining the acceptability and use of recycled plastics in food applications [70].

## 5. Sustainability and Waste Reduction Strategies and Challenges

Broadly, both the US and Canada have recently reinforced the importance of building more sustainable food systems [71,72]. Packaging is an essential element to consider in addressing sustainability [73,74]. It is critical for preserving quality and extending shelf-life to reduce food waste [75], which ultimately influences sustainability. Indeed, changes in packaging that lead to food waste reductions can result in net reductions in environmental impacts, even if the environmental impacts of the packaging itself increase because of a change in material [76]. This consideration is key for specialized products that support vulnerable populations, where the need for safety and preservation of nutrients are paramount. Yet, packaging can also add economic and environmental costs if it is not adapted to meet the needs of specific products, for example if a change in packaging material results in more rather than less product deterioration and food waste [75]. Thus, for specialized products, particular waste reduction strategies must be balanced against the need for packaging materials to meet nutrition integrity, quality and safety requirements, and stringent regulations (Table 4) while still providing the necessary functionality described above.

Globally, the package waste reduction focus has been on adopting a more circular approach through reducing, reusing, and recycling packaging materials [77]. Further perspective on these waste reduction strategies and the potential impact on specialized products is discussed below.

### 5.1. Reducing Packaging Materials

Historically, manufacturers have reduced packaging (and potentially the carbon footprint) by decreasing the weight and materials used in the production of packaging containers [78]. Similarly, manufacturers of specialized products have moved from more traditional packaging in metal cans and glass bottles to lighter weight plastic bottles. Plastic bottles are a predominant form of packaging for these products today and therefore they are the focus of the remainder of this perspective paper. However, source reduction can be limited by manufacturing process requirements. For example, retort processing necessitates the use of sturdy plastic containers due to heat treatment conditions. Such containers are lighter weight than metal or glass but are still heavier than the types of plastic containers that can be used in hot-fill or aseptic processing (Table 3).

### 5.2. Reusing Packaging Materials

In a circular economy, packaging reuse means there is less waste and more product packaging value retained [79]. When considering reusable packaging for specialized products—where the original packaging is collected, cleaned, and then refilled with the same product—safety is a critical issue that must also be evaluated. Several decades ago, Jetten et al. [80] investigated the impacts of reusable plastic packaging on food quality and safety and concluded reuse, i.e., repeated washings of plastic containers, did not significantly influence the chemical, physical, or surface properties of the containers. However, the study only considered standard polyethylene terephthalate (PET) and polycarbonate containers, which are not used in retort systems and do not need to withstand the rigors of this manufacturing process. More recently, a study on reusable plastic sports bottles found that the dishwashing process itself increased migration of plastic-related compounds into drinking water [81]. In products for vulnerable populations, these processes would be contraindicated.

Reusable plastic packaging must also be strong and durable enough to withstand repeated use, which typically requires a greater packaging-to-product-weight ratio than a single-use container. Further, unless there is package standardization, pooling in decentralized supply chains, and decreased overall transport distances, the general environmental impact may not be reduced [82], particularly if the package is not disposed of properly. Thus, a reusable system is not necessarily feasible for all supply chains and packaging [79]. Additional research and development in this area is needed, especially for specialized products. Consideration of relevant regulations is also critical. For example, because of concerns about enhanced sensitivity of infants, the FDA has made different assumptions of exposure assessments and potential increased toxicity data requirements for food contact substances used in infant nutrition product packaging [83,84]. The FDA has also started to regularly impose limitations on food contact material use with infant foods, ingredients, and human milk unless those uses are specifically addressed in an FCN submission [85].

### 5.3. Recycling Packaging Materials

Recycling continues to be the major area of focus for waste reduction, particularly given that in the US, more than half of American trash generated ends up in the landfill [86] and in Canada, most solid waste is sent for disposal rather than diverted through other waste streams [87]. For packaging recycling, cleanliness is a critical issue because recycling may not be a viable option if the packaging is contaminated by food residues [88]. End user knowledge of how to recycle packaging is also important, as Leissner and Ryan-Fogarty identified when studying disposition of infant formula bottles in maternity hospitals [89]. Other factors that can impact recycling include whether the packaging material can actually be recycled and the ability to incorporate post-consumer recycled (PCR) content (reprocessed material made from previously used plastics) into the manufacture of new packaging material.

The recycling of single polymer plastic packaging commonly occurs by mechanical or chemical processes, though economic and ecological issues currently limit large-scale application of chemical recycling [90]. Some plastic packaging, such as PET beverage bottles, generally have a high rate of recyclability. This high rate occurs for multiple reasons, including functioning and distinct/targeted re-collection systems; the chemical nature of PET, which is easily broken down and then rebuilt into virgin polyester; and PET’s very low intrinsic diffusivity for organic molecules (low interaction between packaging material and food). Thus, single polymer PET is viewed as the most promising packaging polymer for closed-loop recycling of post-consumer waste into new food packaging applications—primarily into PET beverage bottles [91]. Although one recent study documented that both PET collection and reprocessing capacity in the US need to increase significantly to meet growing demands for recycling [92].

In contrast, multilayer plastics are generally not recycled into new food packaging because they must be blended with compatibilizers that then yield non-food grade plastic materials [93,94]. This practice is an example of an open-loop recycling process, where the final plastic application is generally of lower quality and is different from the initial application [95]. Compared to single polymer material, multilayer packaging offers improved barrier properties against relevant gases (like oxygen and water vapor) and light that can readily degrade products [96]. For this reason, specialized products are frequently packaged in multilayer plastic bottles and containers (Table 4) to protect critical nutrients from degradation. The use of multilayer plastic limits their ability to be part of closed-loop recycling processes (packaging material is recycled into the same type of new packaging material) until the technologies and infrastructure for such materials advance. Indeed, infrastructure is currently viewed as the primary criterion inhibiting attempts to achieve truly circular food packaging for multilayer plastics [96].

Greater use of PCR plastic in packaging is another way of supporting the circular economy of plastics, yet it too is challenged by multiple factors including contamination from an unintended application by the end-user, cross-contamination from other packaging, and contaminated equipment along the recycling stream. For food contact applications, these challenges are further amplified because contamination from recycled plastics can become a health/safety issue (Table 5) and the critical aspect of chemical safety is often ignored [97]. The FDA’s “main safety concerns with the use of PCR plastic materials in food-contact articles are: (1) that contaminants from the PCR material may appear in the final food-contact product made from the recycled material, (2) that PCR material may not be regulated for food-contact use may be incorporated into food-contact article, and (3) that adjuvants in the PCR plastic may not comply with the regulations for food-contact use. To address these concerns, the FDA considers each proposed use of recycled plastic on a case-by-case basis and issues informal advice as to whether the recycling process is expected to produce PCR plastic of suitable purity for food-contact applications” [98].

A 2021 report on the state of food-grade recycled resin in the US and Canada recently identified several additional barriers to the use of PCR in food contact packaging: insufficient economic drivers, insufficient recycled-content verification requirements that limit benefits to companies, and a limited source of food-grade suitable plastic. Specifically, a lack of supply of suitable feedstock to process into food-grade PCR was viewed as the biggest challenge [104]. One reason for the limited supply may be the stability of certain plastic materials.

Food contact packaging use of PCR is generally focused on PET, since it is one of the most recycled plastics [105]. However, food packaging is also frequently composed of polyolefins, such as the PP and HDPE. Polyolefins are known to degrade after use and recycling [106], which can limit their applicability as a PCR packaging material in common converting processes like thin wall injection molding and extrusion blow molding. Degradation risk limits applicability for use in manufacturing processes requiring high temperature and pressure. In addition, polyolefins have higher potential for contaminant migration than PET [96,106]. Importantly most secondary post-consumer polyolefins are currently not permitted for use in food contact materials [106]. Many specialized products use plastic bottles made from the polyolefin PP (Table 3) and all the above are serious concerns when considering PCR packaging mandates. The significant need for manufacturing process innovation, development, and research for polyolefin PCR to reach commercial scale of use while still ensuring product safety and quality cannot be overstated, particularly when considering specialized products for vulnerable populations.

### 5.4. Biobased Packaging and Compostability

Greater public awareness of the environmental challenges related to conventional plastic materials and consumer pressure for improved sustainability have triggered the development of biobased, biodegradable (compostable) food packaging materials [107]. These packaging materials can be eco-friendly and easier to recycle or compost. Some potential applications for specialized products include the use of biobased materials in lids and scoops for powdered infant formulas [108]. However, biobased materials also have limitations including increased manufacturing costs, potentially more limited physical protection/mechanical strength (important for product transportation and storage), potential incompatibility with certain food products, and most importantly a disparate recycling infrastructure that at present is not aligned with effective recycling of these materials [1]. In addition, current biobased materials have relatively poor heat stability compared to traditional plastics [109], which limits their applicability in packaging for specialized products that use retort and hot-fill manufacturing processes with high temperature requirements to reach commercial sterility.

## 6. Recycling and Sustainability Policy Framework

A 2019 analysis using data from World Bank’s “What a Waste” global database calculated special waste and regular municipal solid waste per capita to identify the world’s largest producers of waste. Canada ranked as the largest producer and the US ranked third [110]. Both countries are developing policies and programs to reduce solid waste and increase recycling.

### 6.1. United States

For most countries in the world, including the US, policy-making related to waste management and recycling has primarily been the purview of state and local governments [111]. However, there is evidence of potential for increased US federal government engagement. Recently, international trade decisions, fluctuating commodity markets, and shifting public opinion have renewed federal interest in the national recycling infrastructure [112]. The 117th US Congress (2021-22) introduced over 20 bills on topics including increasing recycling, improving recycling accessibility, and reducing plastic waste [113]. A 2020 US Government Accountability Office (GAO) report identified 5 cross-cutting challenges that affect the American recycling system: (1) contamination of recyclables; (2) low collection of recyclables; (3) limited market demand for recyclables; (4) low profitability for operating recycling programs; and (5) limited information to support decision making about recycling. The report recommended these as important focus areas for federal efforts [114].

Further, even though the US federal government does not directly regulate waste, the Environmental Protection Agency (EPA) does have the authority to control hazardous waste under the 1976 Resource Conservation and Recovery Act (RCRA) and its subsequent amendments [115]. As part of this work the EPA also develops policies and programs that encourage source reduction and beneficial reuse, bolster the nation’s recycling infrastructure, and increase municipal solid waste recycling/composting. In particular, EPA has been leading recycling efforts by bringing together key stakeholders to identify specific actions to address the US recycling system’s challenges. Most recently, the EPA set a National Recycling Goal to increase the recycling rate to 50% by 2030 [116]. In support of this goal, the EPA has developed a *National Recycling Strategy* that outlines 5 strategic objectives, including “Enhance Policies and Programs to Support Circularity,” which aims to “increase coordination, availability, and accessibility of information on recycling programs and policies at the federal, state, tribal and local levels” [117].

Thus, even with increased federal interest, there is recognition that state and local governments will continue to play a primary role in public policy on waste reduction and recycling. The National Conference of State Legislatures reported in 2020 that at least 27 states and the District of Columbia had at least one mandatory recycling requirement and, in the 2020 legislative sessions, over 600 bills were introduced with a primary focus on better understanding, investing in, and improving recycling [112]. New state-level sustainability legislation and regulation seemed to stall during the height of the COVID-19 pandemic but has been increasingly active in 2022. These policy actions have included extended producer responsibility (EPR) bills, which require manufacturers to take ownership of the environmental impacts of their products throughout the product lifecycle and pay fees accordingly, and PCR bills, which set minimum requirements for PCR content in packaging materials. EPR bills increase the cost of manufacturing and ultimately product costs, which can be an added burden on vulnerable populations. PCR mandates can pose particular challenges for specialized products, potentially constraining access to these products and increasing costs for the vulnerable populations who need them. As explained earlier in the paper, these products are packaged in multilayer plastic bottles and containers that curb inclusion in closed-loop recycling processes, while limits in supply of food-safe materials, material instability, and contaminant migration risk often restrict use of PCR.

### 6.2. Canada

Canada has moved to take a strong federal role in waste management. For over 10 years, there has been a Canada-wide Action Plan for Extended Producer Responsibility [118]. While the implementation of the plan is left to the jurisdictional authority of each provincial government, the plan provides “common coordinated policies and commitments for government action and common key elements for building producer responsibility through the adoption of EPR approaches to identified priority products”. For example, the government of Ontario recently announced the expansion of its EPR program to standardize the materials that can be recycled and shift full responsibility for the cost and operation of the program to product and packaging producers [119]. In support of such provincial and territorial EPR efforts, the Government of Canada is proposing to establish a federal plastics registry and requiring producers to report on plastics in the Canadian economy [120].

In 2018, ocean health and plastic pollution were made priorities for Canada’s 2018 G7 presidency, and federal, provincial, and territorial environment ministers agreed to push forward on a Canada-wide zero-plastic-waste strategy [121]. In June 2022, Canadian regulations were published prohibiting the manufacture, import, sale, and export of 6 specific single-use plastic items [122]. In addition, the federal government has proposed that plastic packaging in Canada contain at least 50% recycled content by 2030 [123]. Specialized products will be uniquely challenged to meet this proposed implementation timeline because, as explained, viable PCR plastic packaging alternatives do not currently exist, which could potentially limit access for the vulnerable populations who require these products. The federal government has also proposed prohibiting use of the chasing-arrows symbol on plastic packaging unless 80% of Canada’s recycling facilities accept the packaging and have reliable end markets for the packaging [124].

## 7. Innovation and Policy Development for Sustainability

Some emerging food packaging innovations may offer promise for specialized products in the future, but currently the products will continue to be uniquely challenged by functional food packaging requirements for quality/safety and processing demands and by policy development for sustainability.

### 7.1. Implications for Emerging Innovation

Spurred by the need for increased sustainability, packaging innovation seeks to meet the demands of both product users and manufacturers [125]. Our paper has described how some innovations, specifically the use of PCR and biobased materials in packaging, are currently limited in their functional applicability and availability for packaging of specialized products. More research and development as well as changes in the recycling infrastructure are needed to bring such materials to scale while maintaining product quality and safety.

Other areas of packaging innovation include active and intelligent packaging technologies, which aim to deliver higher quality and safer products and potentially reduce product waste. Active packaging acts on the physical environment surrounding the product to increase shelf-life while intelligent packaging allows for tracking and/or monitoring of the condition of the packaged product [126].

Active food packaging is often classified according to the function of the additive it contains, most commonly those with antioxidant or antimicrobial activity. Recent research includes studies focused on natural polymers and pigments, particularly those that can be extracted from food industry waste or by-products which both add value and reduce production costs [127]. If such innovations could be further studied for feasibility of use in products for vulnerable populations and developed to withstand the manufacturing rigors of specialized products, the innovations would bring a welcome advantage.

Overall, research on active packaging for specialized products is still in the early stages, but there is some evidence of potential benefit, for example in helping minimize the oxygen in packaged powdered infant formula to preserve quality and limit nutrient degradation [128]. Bringing such applications to scale could help safeguard the quality and nutrition integrity of specialized products and benefit the vulnerable populations who rely on these products.

Specialized products can benefit from development of other emerging packaging technologies, too. Routine quality and safety monitoring are integral to manufacturing processes, but such monitoring is often limited once finished products enter distribution systems. The strength of intelligent packaging is in its ability to communicate and continuously provide information about both products and their packaging integrity as they journey through retail supply chains [126]. Intelligent packaging can be achieved via 3 main technologies: indicators, sensors, and data carriers [129]. By helping support quality and safety, this packaging can potentially reduce waste. However, the current generation of intelligent packaging that leverages these technologies to provide continuous monitoring throughout the supply chain has mainly been prepared on a small scale, within research facilities, and without consideration of true manufacturing costs. More research and development are required to reduce the costs of materials and processing operations used to create intelligent packaging materials and thus make them more commercially viable and applicable to a wider range of products [130].

In a positive move, manufacturers of specialized products are already taking advantage of some intelligent packaging technology, such as using quick response (QR) codes to provide greater transparency on ingredients, quality, sourcing, and product movement through the supply chain [131] and to help ensure product authenticity [132]. In the future, further research and advancement of intelligent packaging may help users of specialized products in other ways too, such as sending notification if a product is expired, providing additional information beyond what can be included on the product label (e.g., detailed product preparation, use, and storage information), and how a product’s packaging supports sustainability and may be recycled.

### 7.2. Implications for Policy Development for Sustainability

In the US, and to a lesser degree in Canada, legislation and regulation impacting packaging continues to be asynchronized, with states or provinces working independently. Examples include state/province-specific requirements for recycling symbols/instructions on product packaging and/or labels, bans of various types of single use plastic, PCR packaging requirements with different set percentage levels by a certain time, and EPR requirements that may penalize certain packaging volumes or materials.

The incongruency in state/provincial policies remains a challenge for all food manufacturers who distribute their products nationally and across the US/Canada border. It can be especially difficult for manufacturers of specialized products who must maintain inventories to meet critical patient needs and cannot idle manufacturing lines to retool for a specific state/province mandate or create a unique product package or label for one state/province alone. Recent PCR legislation signed into law in New Jersey does include exemptions for certain specialized products, including medical foods, FSDUs, and infant formulas [133]. However, without such exemptions, evolving and incongruent state/provincial packaging sustainability legislation/regulation continues to pose challenges for vulnerable populations. Federal standardization would help resolve some of these issues.

Many different organizations and coalitions have put forth proposals on the policy actions needed to fix broken recycling systems. In the US, one such diverse advisory is the Recycling Leadership Council, with over 20 groups representing “consumer-facing industries, packaging suppliers, non-governmental organizations, and academic thought leaders.” The Council recently released a *Blueprint for America’s Recycling System*, calling for policy action in 3 areas: data collection, system standardization and harmonization, and financing and end-market development. The *Blueprint* included federal policy recommendations that echo the need for uniform, national recycling standards to help inform state action on packaging and recycling policies like EPR or PCR requirements [134].

Similarly in Canada, more than 70 businesses, organizations, and governments came together through the Canada Plastics Pact to release *Roadmap to 2025*, a plan representing a “shared vision for a circular economy for plastics packaging and a collaborative action plan to drive tangible change by 2025”. The *Roadmap* identified 3 strategic priorities: reduce, reuse, collect; optimize the recycling system; and use data to improve the whole system. It also emphasized ensuring government policy is in place, well-designed, and creates standard definitions and measurement practices [135]. However, to date neither the US *Blueprint* nor Canadian *Roadmap* consider the potential policy impacts on products for vulnerable populations and the critical need to ensure the continued safety, quality, cost stability, and access for specialized products.

## 8. Conclusions

The literature is limited regarding the role and importance of functional and sustainable packaging for specialized products for vulnerable populations, such as infants and young children, those who are ill, and older adults. This paper summarized the regulatory categorization, the role of packaging, and processing considerations/food contact regulations in the selection of functional packaging materials for specialized products used by vulnerable populations. Packaging materials for specialized products must withstand requisite sanitization/sterilization and processing/heat treatment conditions, keep products safe, and protect against potential toxic chemical migration from packaging materials to the food, as well as maintain product nutrient levels to support the nutrition of vulnerable populations. This places both very high requirements and expectations on the role that packaging plays for specialized products.

Furthermore, described were the challenges these products face relative to waste reduction strategies and North American legislative/regulatory actions, as well as possible implications for innovation and policy development. EPR bills increase the cost of manufacturing and ultimately product costs, which can be an added burden on vulnerable populations. PCR mandates can also increase costs and pose particular challenges for specialized products, as these products are packaged in multilayer plastic bottles and containers that curb inclusion in closed-loop recycling processes. Additionally, limits in the supply of food-safe materials, material instability, and contaminant migration risks can be factors that often restrict the use of PCR materials.

While there will likely be opportunities for improving the sustainability of packaging for specialized products, the functional developments and applications of emerging technologies may not come as quickly or as easily as they may for other food products, because of the unique nature of specialized products and the populations served. Without adequate consideration of such barriers and paths for exemptions, evolving and incongruent state/provincial packaging sustainability legislation/regulation will continue to pose challenges for products supporting vulnerable populations and potentially limit access to the critical nutrition products such groups need to thrive.

## Figures and Tables

**Table 1:** Regulatory definitions for specialized product categories in the United States.

Regulatory Category	Statute/Regulation	Definition
Food for Special Dietary Use (FSDU)	21 CFR 105.3	“The term special dietary uses, as applied to food for man, means particular (as distinguished from general) uses of food, as follows: (i) Uses for supplying particular dietary needs which exist by reason of a physical, physiological, pathological or other condition, including but not limited to the conditions of diseases, convalescence, pregnancy, lactation, allergic hypersensitivity to food, underweight, and overweight; (ii) Uses for supplying particular dietary needs which exist by reason of age, including but not limited to the ages of infancy and childhood; (iii) Uses for supplementing or fortifying the ordinary or usual diet with any vitamin, mineral, or other dietary property. Any such particular use of a food is a special dietary use, regardless of whether such food also purports to be or is represented for general use” [14]
Medical Food	21 USC 360ee(b)(3)	“A food which is formulated to be consumed or administered enterally under the supervision of a physician and which is intended for the specific dietary management of a disease or condition for which distinctive nutritional requirements, based on recognized scientific principles, are established by medical evaluation” [11]
Infant Formula	21 USC 321(z)	“A food which purports to be or is represented for special dietary use solely as a food for infants by reason of its simulation of human milk or its suitability as a complete or partial substitute for human milk” [15]
Exempt Infant Formula	21 CFR 107.3	“An infant formula intended for commercial or charitable distribution that is represented and labeled for use by infants who have inborn errors of metabolism or low birth weight, or who otherwise have unusual medical or dietary problems” [16]

**Table 2:** Regulatory definitions for select * specialized product categories in Canada.

Regulatory Category	Regulation	Definition
Food for Special Dietary Use (FSDU)	FDR B.24.001	“Food that has been specially processed or formulated to meet the particular requirements of a person (a) in whom a physical or physiological condition exists as a result of a disease, disorder or injury, or (b) for whom a particular effect, including but not limited to weight loss, is to be obtained by a controlled intake of foods” [22]
Formulated Liquid Diet (FLD)	FDR B.24.001	“A food that (a) is sold for consumption in liquid form, and (b) is sold or represented as a nutritionally complete diet for oral or tube feeding of a person described in paragraph (a) of the definition ‘food for special dietary use’” [22]
Meal Replacement (MR)	FDR B.24.200	“A formulated food that, by itself, can replace one or more daily meals” [23]
Nutritional Supplement(NS)	FDR B.24.201	“A food sold or represented as a supplement to a diet that may be inadequate in energy and essential nutrients” [23]
Human Milk Substitute (Infant Formula)	FDR B.25.001	“Any food that is labelled or advertised (a) for use as a partial or total replacement for human milk and as intended for consumption by infants, or (b) for use as an ingredient in a food referred to in paragraph (a)” [24]
Human Milk Fortifier (HMF)	FDR B.25.001	“A food that (a) includes at least one added vitamin, mineral, nutrient or amino acid, and (b) is labelled or advertised as intended to be added to human milk to increase its nutritional value in order to meet the particular requirements of an infant in whom a physical or physiological condition exists as a result of a disease, disorder or abnormal physical state” [24]
Natural Health Product (NHP)	Natural Health Product Regulations (NHPR)	“A substance … that is manufactured, sold or represented for use in (a) the diagnosis, treatment, mitigation or prevention of a disease, disorder or abnormal physical state or its symptoms in humans; (b) restoring or correcting organic functions in humans; or (c) modifying organic functions in humans, such as modifying those functions in a manner that maintains or promotes health” [20]

* As discussed in this paper.

**Table 3:** Manufacturing processes used for producing specialized products for vulnerable populations [30,49,50,51].

Manufacturing Process	Description	Advantages/Disadvantages	Product Examples	Common Packaging Materials *
Retort	Non-sterile product filled into hermetically sealed, non-sterile packaging that is bulk loaded into retort bin; bin subjected to high heat/pressure for extended time to commercially sterilize product/packaging	**Advantage** Retort is a well-known/researched food manufacturing process **Disadvantages** Very high temperature/pressure for extended time can impact product quality (ex. nutrition, organoleptics) and degrade some plastic packaging materials Limits product protein content Throughput can be slower than other manufacturing processes	Low-acid foods (pH > 4.6, water activity > 0.85) including liquid forms of infant formulas, oral nutrition supplements	Metal Glass Multilayer composite drink carton Semi-rigid plastic made from materials able to withstand high temperature (ex. polypropylene (PP)) Multilayer flexible pouches
Hot-fill	Heated, commercially sterile product filled into non-sterile packaging that is sealed, held at high temperature for short time (to commercially sterilize inner surface of package), and then immediately cooled	**Advantages** Product/packaging held at lower temperature/for less time vs. retort, so impact on product/packaging is reduced Throughput can be faster vs. retort **Disadvantages** High temperature can still degrade some plastic packaging materials Heavier weight packaging required vs. aseptic; less sustainable and higher costs	High-acid foods (pH < 4.6) including oral electrolyte solutions	Glass Semi-rigid plastic able to withstand production line’s fill temperature (PP and hot-fill polyethylene terephthalate (PET))
Aseptic	Product and packaging commercially sterilized, product filled into packaging in commercially sterile environment	**Advantages** Product/packaging not exposed to high temperature/pressure for extended time, reducing impact on product quality and packaging materials (provides greater flexibility in packaging) Throughput can be faster vs. retort Higher product protein levels Lighter weight packaging is more sustainable and lower cost **Disadvantage** Manufacturing process set-up and materials can be more expensive vs. other manufacturing processes	Low- and high-acid foods including liquid forms of infant formulas, oral nutrition supplements, oral electrolyte solutions	Metal Glass Multilayer composite drink carton Semi-rigid plastic bottles including PET and HDPE Multilayer flexible laminations (stickpacks, pouches, etc.)
Powdered	Product prepared, evaporated/dried, then product filled into package	**Advantage** Product/packaging not exposed to high temperature/pressure for extended time, reducing impact on product quality and packaging materials (provides greater flexibility in packaging) **Disadvantage** Product is not commercially sterile	Infant formulas, oral nutrition supplements, oral electrolyte solutions	Metal Fiber-based composite Multilayer flexible laminations (stickpacks, pouches, etc.) Semi-rigid plastic

* All packaging for oxygen-sensitive products requires excellent barrier properties (i.e., multilayer materials).

**Table 4:** Packaging requirements and potential quality/safety benefits and risks of effective vs. ineffective packaging materials for specialized products for vulnerable populations.

Packaging Requirement	Potential Quality/Safety Benefits from Effective Packaging Materials	Potential Quality/Safety Risks from Ineffective Packaging Materials
Material Performance: Withstand requisite processing and heat treatment conditions	Maintains packaging integrity throughout manufacturing process	Packaging degrades or fails during manufacturing resulting in product loss *^,†,‡^
Packaging Performance: Withstand rigors of supply chain and distribution process	Maintains packaging integrity during transportation, warehousing, retail sale, and consumer storage/use	Packaging is damaged during distribution, resulting in unsaleable product ^†,‡^
Shelf Life: Maintain commercial sterility	Keeps product shelf-stable and safe to consume for an extended period without refrigeration	Biological protection not maintained, increasing product susceptibility to spoilage and risk for foodborne illness ^†,‡,§^
Barrier Requirements: Maintain nutrition value	Packaging barrier (light, oxygen, moisture) properties ensure product nutrient levels meet label claims throughout product shelf life	Packaging lacks adequate barrier properties, allowing nutrients to degrade and resulting in product not able to adequately meet consumer nutrition needs and label claims ^†,§^
Food Safety: Use is safe for food contact	Proper material selection limits migration of unintended, unsafe contaminants from the packaging materials to the product during processing and throughout product shelf life	Unintended contaminants, compatibilizers, and/or byproducts that are restricted from food contact use migrate to product, jeopardizing consumer health and ability to meet food safety regulations *^,^^†^

* Risk of materials unable to withstand high temperatures (such as polyethylene terephthalate or PET). ^†^ Risk of post-consumer resin (PCR). ^‡^ Risk of biobased materials. ^§^ Risk of single polymer non-barrier material.

**Table 5:** Potential contaminants of concern and possible health issues related to use of recycled plastics in food packaging materials.

Potential Contaminants of Concern	Possible Health Issues
Phthalates (including anti-androgenic phthalates dibutyl phthalate (DBP) and diisobutyl phthalate) [99] Bisphenol A (BPA) Bisphenol S (BPS) among others including bisphenol A diglycidyl ether and derivatives (BADGE) [60] Antimony (Sb)—depending on treatment and storage conditions [100] Heavy metals including Chromium, Lead (Pb), Antimony (Sb), Nickle (Ni) [101] Alcohols, esters, ketones, fragrance, and flavor compounds [102]	Endocrine disruptors—potential implications for reproductive systems, metabolic disorders including diabetes and obesity [103]

## Data Availability

Not applicable.

## Abbreviations

CFR	Code of Federal Regulations
EPR	Extended Producer Responsibility
FAP	Food Additive Petition
FCN	Food Contact Notification
FCS	Food Contact Substance
FD&C Act	Food Drug and Cosmetic Act
FDA	US Food and Drug Administration
FDR	Health Canada’s Food and Drug Regulations
FLD	Formulated Liquid Diet
FSDU	Food for Special Dietary Use
GMP	Good Manufacturing Practice
GRAS	Generally Recognized as Safe
HDPE	High Density Polyethylene
HPFB	Health Products and Food Branch
LDPE	Low Density Polyethylene
LONO	Letter of No Objection
MR	Meal Replacement
NHP	Natural Health Product
NHPR	Natural Health Product Regulations
NS	Nutritional Supplement
PCR	Post consumer recycled
PE	Polyethylene
PET	Polyethylene terephthalate
PP	Polypropylene
USC	United States Code
USDA	United States Department of Agriculture
